# The first description in the literature, of a complication during the extraction of a retrograde expandable intra-medullary nail in three patients

**DOI:** 10.1016/j.tcr.2016.03.006

**Published:** 2016-04-13

**Authors:** K. Tilkeridis, T. Tzatzairis, M. Tryfonidis, D.S. Elliot, R.B. Simonis

**Affiliations:** a Department of Orthopaedic Surgery, Medical School, Democritus University of Thrace, University General Hospital of Alexandroupolis, 68100 Alexandroupolis, Greece; b General Hospital of Nicosia, Cyprus; c Dept Orthopaedics, Ashford & St Peter's Hospital NHS Trust, Chertsey, United Kingdom

**Keywords:** Ankle fracture, Intramedullary nail, Fixion nail, Retrogade nail, Tibia fracture, Complication

## Abstract

Ankle fractures in elderly people are low-energy fractures characterised by fragility. In the majority, they are unstable and challenging to manage.

Retrograde expandable intra-medullary nails (Fixion®, Biomet Merck Limited) inserted through the calcaneum across the sub-talar and ankle joints into the tibia have been successfully used in the treatment of fragility fractures and non-unions of the distal tibia and ankle, where the use of an antegrade locked nail would not provide adequate fracture stability for union.

Primary fracture management involves removing the nail at least 3–4 months after radiological check. In cases of treatment of non-unions a longer treatment period is often required before removal of nail is considered.

We present three patients where breakage of the Fixion® nail during surgery caused problems in nail extraction.

## Introduction

After the wrist and hip, the third most common fracture in elderly people is at the ankle [1]. These, low-energy, fractures are characterised by fragility and occur mostly in elderly osteoporotic women [2], [3], [4]. In the majority, they are unstable and challenging to manage [5].

Retrograde expandable intra-medullary nails (Fixion®, Biomet Merck Limited) inserted through the calcaneum across the sub-talar and ankle joints into the tibia have been successfully used in the treatment of fragility fractures and non-unions of the distal tibia and ankle, where the use of an antegrade locked nail would not provide adequate fracture stability for union [6]. The advantage of this system is the immediate mobilisation of the patient and the decreased risk of bone or wound problems [7].

Primary fracture management and our unit's policy too, involve removing the nail at least 3–4 months following clinical and radiological confirmation of fracture union [6]. In cases of treatment of non-unions a longer treatment period is often required before removal of nail is considered.

We present three patients where breakage of the Fixion® nail during surgery caused problems in nail extraction. Technical difficulties in removal of the Fixion® expandable intra-medullary nail have not been reported to date and it presents a difficult situation to manage as the extraction kit does not include appropriate instrumentation to remove a broken nail.

## Case one

**Fig. 1:**
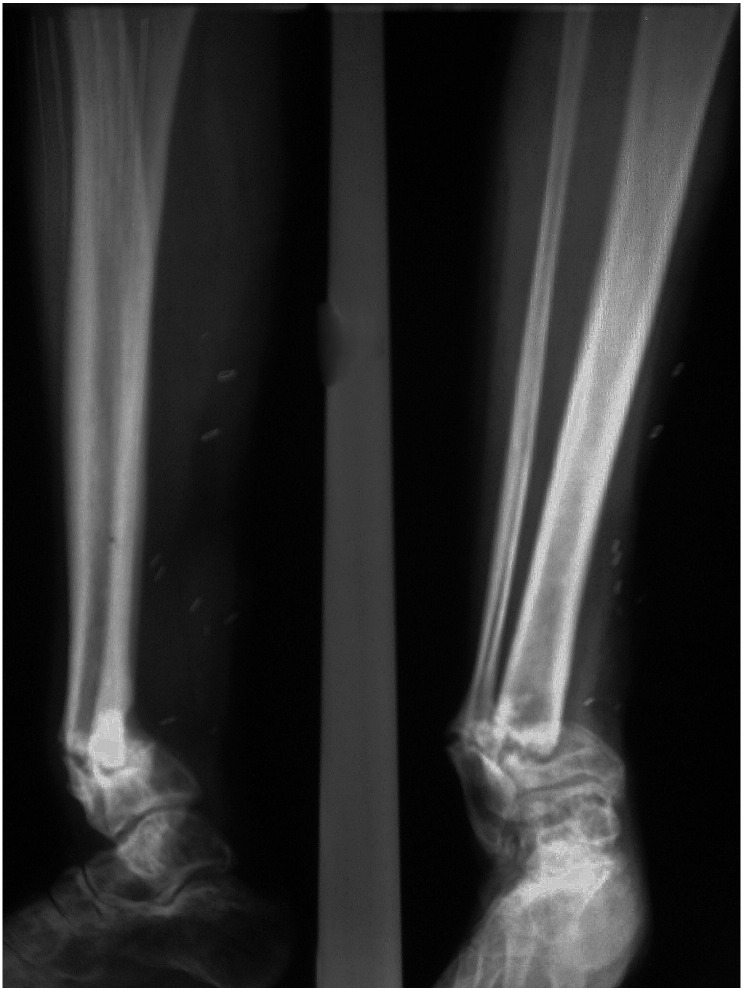
Right distal tibial non-union fracture (deviated into varus deformity).

**Fig. 2:**
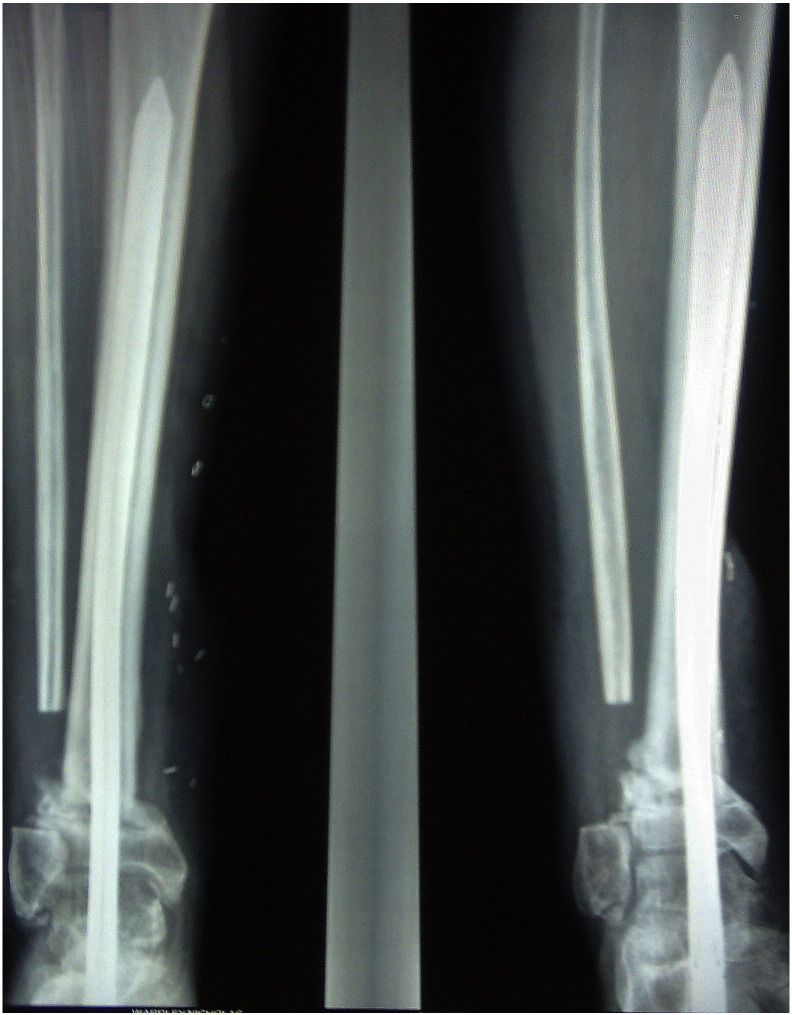
Non-union fracture stabilization using a retrograde expandable nail.

**Fig. 3:**
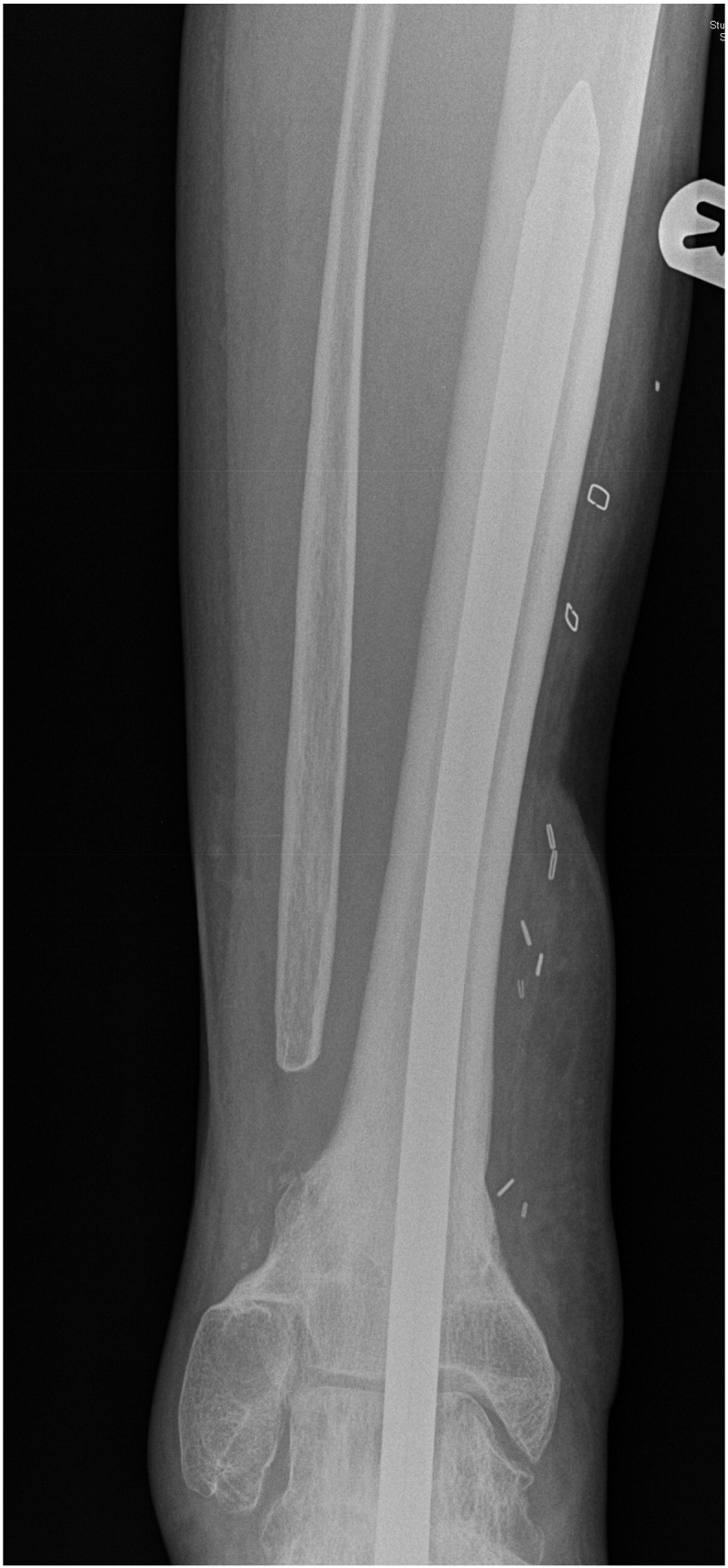
United distal tibial fracture within 6 months of the operation.

A 35 year-old male was referred to our unit with a right distal tibial non-union, which had progressively deviated into varus deformity. He had initially sustained a high-energy, multifragmentary compound (Gustilo–Anderson Type 3A) fracture in a road traffic accident 4 months earlier. After successful treatment of the soft tissue injuries he had been managed in a below-knee, weight-bearing Sarmiento cast. His non-union (Fig. 1, Fig. 2) was stabilised with a retrograde 10 mm × 340 mm expandable nail. Regular clinical and radiological follow-up revealed that the non-union had solidly united within 6 months of the operation (Fig. 3). The patient was able to walk comfortably and return to work as a carpet fitter.

**Fig. 4:**
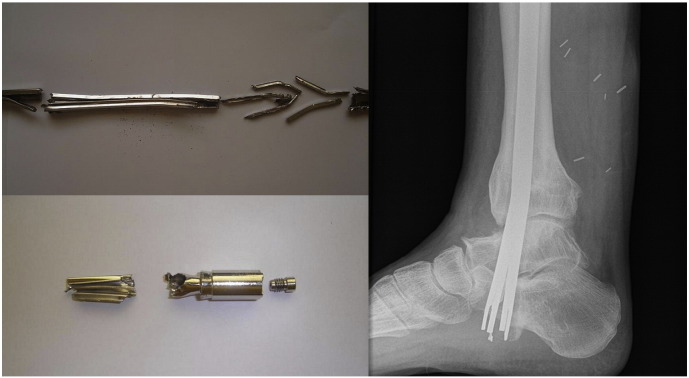
Fractured nail at the junction between the valve and the metal fins.

Nine months after insertion of the nail there were radiological appearances suggestive of calcaneal resorption around the neck of the nail, therefore we recommended removal of the nail. This was attempted under general anaesthesia using the kit and technique described by the manufacturer. After deflation of the nail, attempts were made to “back-slap” the nail out of the tibia. This failed as the nail fractured at the junction between the valve and the metal fins (Fig. 4). Further attempts to remove the nail using grabbers and a mole wrench were unsuccessful and the procedure was abandoned.

**Fig. 5:**
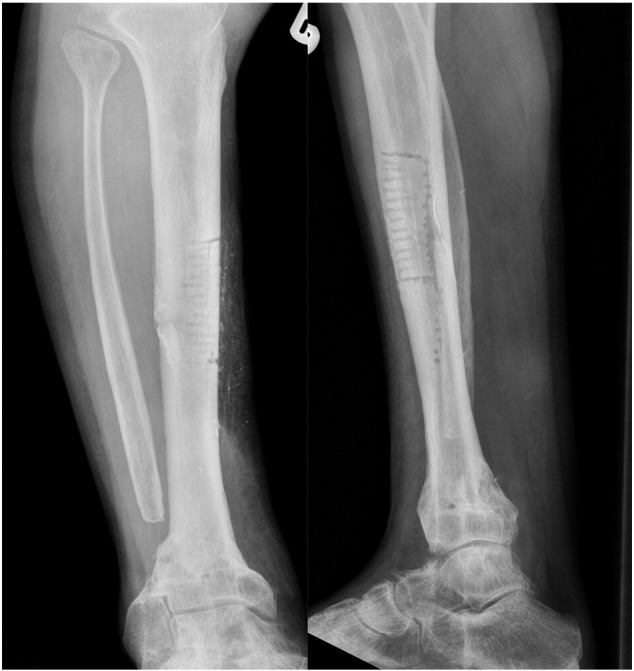
Nail removal through a 6 cm x 2 cm antero-medial tibial window.

The patient returned to theatre for a second attempt. Initial attempts at removal through the entry point using grabbers and large diameter crown drills were unsuccessful as the broken end of the nail had split, with the fins separating, like an inverted cone. A further attempt at pushing the nail down using an antegrade nail was also unsuccessful. Eventually, the nail was removed piecemeal through a 6 cm × 2 cm antero-medial tibial window using a Midas Rex® burr (Medtronic Ltd., Watford) (Fig. 5). The tibia was protected in a below-knee walking cast for 6 weeks and the patient made an uneventful recovery with no further complications.

## Case two

A 67 year-old female was referred with a right distal tibial non-union with progressive valgus deformity following a fragility fracture five months previously. It was initially treated with locked antegrade intra-medullary nailing.

The initial nail was removed and an expandable retrograde nail was inserted. Two months later an Ilizarov frame was applied over the nail to provide further compression. The non-union united and the frame were removed three months later. The nail was left for six more months in order to allow further consolidation of the non-union.

The first attempt to remove the expandable nail was made ten months after insertion. The standard technique to remove the nail was attempted, but again the procedure failed because the nail fractured at the junction between the valve and the fins. Further attempts to remove the nail with grabbers at that time were unsuccessful and the procedure was abandoned. Two months later, a second attempt was undertaken as the patient was complaining of ankle pain. The track from her primary nail was re-opened from the proximal end and the expandable nail was successfully pushed out using an antegrade nail. This procedure passed without incident and she made a good post-operative recovery.

## Case three

A 73 year-old male presented to our unit with a multifragmentary Pilon fracture and was treated with primary retrograde expandable Fixion® nail. Regular follow-up revealed good union at 22 weeks and the patient was able to walk without significant pain. Although we recommended the removal of the nail, the patient didn't consent on it.

**Fig. 6:**
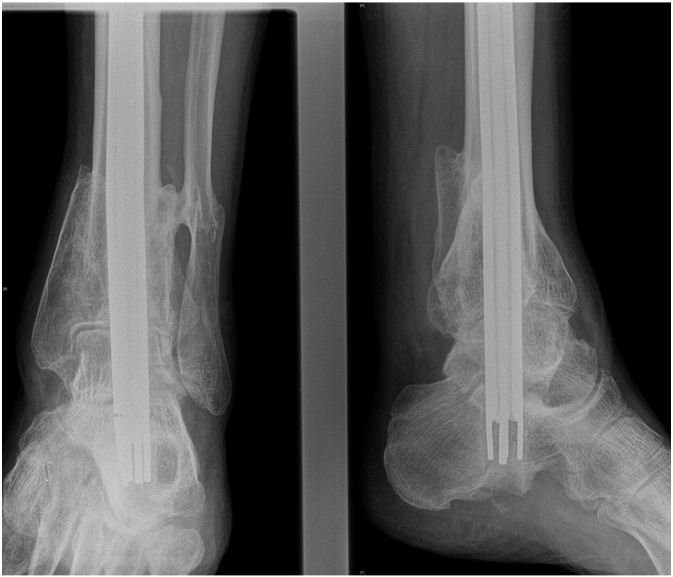
Fractured nail at the junction of the valve and fins during its extraction.

Two and a half years later the patient requested removal of the nail because of chronic heel pain presumably due to slight prominence at the bottom end of the nail. Using the standard extraction technique an attempt to remove the nail was performed but resulted in fracture of the nail at the junction of the valve and fins (Fig. 6). The end cup and valve were removed but attempts to remove the remaining part of the nail with grabbers failed and the procedure was abandoned.

The patient was advised to keep the nail remnant unless it becomes symptomatic. He is still under follow-up and currently asymptomatic.

## Discussion

The Fixion expandable nail is a stainless steel sealed tube made up of four longitudinal bars and can be easily introduced in a similar way to an unreamed nail. Having a reduced initial diameter, for easier insertion, it is expanded with saline solution using a hydraulic pump. The four longitudinal bars are forced against the cancellous and cortical bone and the nail adapts itself to the contours of the medullary canal. The nail expands up to 160% of its original diameter and provides axial and rotational stability only by self-expansion [8]. This system has been used successfully in humeral and femoral fractures and has even been used in treating fragility fractures of the ankle. It eliminates the need for interlocking screws and reaming of the medullary canal, offers a minimally invasive procedure for intra-medullary fixation, and biomechanically causes the nail to assume the hourglass shape of the medullary canal by its abutment to the medullary walls [6], [9], [10], [11], [12], [13]. Since no interlocking screw fixation is needed with the Fixion nail, there is an important decrease in fluoroscopy and operative time. It is simple to apply and can be effortlessly removed by deflation. It can be inserted through one entry point, blind power tools are not used and the possibility of nerve damage, noted in interlocking nails, is reduced [14], [15]. One more major advantage is that Fixion nail does not require reaming, which increases intramedullary pressure and may lead to pulmonary and cardiac phenomena, it can be safely used in indicated to polytraumatized patients with chest injuries [16], [17].

Reported complications associated with the use of the expandable nail include delayed and non-union, fracture shortening due to inadequate axial stability, nail protrusion at the knee, compartment syndrome, fracture propagation after nail expansion, distal mal-alignment, and the requirement of a secondary procedure [8], [18], [19]. Removal of hardware up until now has been uneventful and straight-forward [8]. In the published series of retrograde insertion technique there were no reported ankle or subtalar short-term problems compared to long-term complications that include arthritis of the ankle and subtalar joint. The advantage of using an expandable nail is that the stability on the site of the fracture is sufficient to allow instant unrestricted weight-bearing without a cast [6].

In all three cases the nail broke at the junction of the valve and fins which separated as an inverted cone. This particular segment of the nail was located near the (mobile) subtalar joint and was exposed to continuous stress and strain for more than nine months during patient mobilisation. Moreover this particular segment of the nail seems to be very thin and probably more vulnerable to metal fatigue. Another factor may be a possible bone in-growth around the fins of the nail, which could interfere with both deflation and its passage out of the medullary cavity.

In the second case, the circular frame was applied using radiographic control to ensure none of the wires damaged the nail during their insertion. Although no damage was noticed during Ilizarov's application or removal, we accept though that this may have contributed to difficulties in nail extraction.

We believe that early removal of this nail should be considered when possible in order to avoid advanced metal fatigue and potential nail fracture during extraction. We manage to extract the nail, within the first 6 months, from patients who were treated for fragility fractures with no complication. However, early removal of the expandable nail may not always be advocated, particularly when dealing with distal tibial non-union or fragility fracture. Moreover, the manufacturing company may need to revisit the design of the nail in order to improve the strength at the junction of the valve and fins. It may also be beneficial to include appropriate instrumentation in the extraction kit for the event of nail fracture.

## Conclusion

Retrograde expandable intra-medullary nails are an effective method of treatment for distal tibial fragility fractures and non-unions. We recommend stabilisation of these fractures using an expandable intramedullary nail as it has a low risk of complications and allows an immediate return to full weight-bearing. Our recommendation is the, as soon as possible, removal of the nail within 6 months of the insertion to reduce the risk of nail fracture at extraction. In case of nail's retention for more than one year it is advised not to attempt its removal, on condition that no complication is encountered.

## Conflict of interest

All authors have no conflict of interest to declare.
